# Editorial Notes

**Published:** 1942

**Authors:** 


### Editorial Notes

**Return of Colonel Clarke.**

The return of Colonel R. C. Clarke to civil life and practice is a matter of great satisfaction to his colleagues.

His military career dates back to 1903 when he joined the Bristol Rifles as a private. He received his commission as combatant officer in the old volunteer force and in 1908 became a territorial. After obtaining his medical qualification, he joined the R.A.M.C. and served for many years as Regimental Medical Officer to the 4th Gloucesters, with whom he went to France in 1915 with the other units of the 48th South Midland Division.

After a period of service as Regimental Medical Officer he joined No. 19 C.C.S., where he was in charge of the medical wards.

For his outstanding services in the last war he received the O.B.E., military division.

At the outbreak of war in 1939 he was still on the active list of the Territorial Army with the rank of Colonel. After a period of service at home, he proceeded to the Middle East in command of a General Hospital. A troublesome illness whilst abroad automatically deprived him of his command, and he returned to England. On his arrival in England, although completely restored to health, the War Office decided to place him on the retired list. Since he appears to be in command of excellent health and his vigour is undiminished, his value in civil practice and in the hospital work of Bristol cannot be over estimated.

**Royal Naval Hospital.**

An interesting series of demonstrations and lectures was arranged at the Royal Naval Auxiliary Hospital for the winter months. They were held in the Recreation Hut every fortnight on Fridays at 4.30 p.m. Members of the Bristol Medico-Chirurgical Society were invited to attend.

The programme was as follows: 4th December, Surgeon-Captain Macdonald Critchley, R.N.V.R.: "Medical Aspects of Shipwreck." 18th December, Clinical Meeting: Demonstration of cases.

1st January, Surgeon-Commander J. Green, R.N.V.R. : " Bacteriological Aspects of Acute and Sub-Acute Rheumatism " (title and date to be confirmed). 15th January, Clinical Meeting : Demonstration of cases. 29th January, Surgeon-Captain Lambert Rogers, R.N.V.R. : " Head Injuries." 12th February, Clinical Meeting : Demonstration of cases. 26th February, Surgeon-Captain Desmond Curran, R.N.V.R. : " Depressions."

These clinical afternoons have been very greatly appreciated. Our Naval colleagues have made themselves an integral part of the Medical life of Bristol.

**Anæsthesia for Shocked Patients.**

IN our Spring, 1942, issue a discussion on " Anæsthetics " was reported ; but there was little mention made of the special difficulties which attend the anæsthetization of shocked patients. The Ministry of Health has just issued an " instruction " prepared by a sub-committee of the Medical Research Council and the Consultant Adviser in Anæsthetics. It appears to be of great importance to all medical officers who may be called upon to give anæsthetics in an emergency. In the first instance, naturally an experienced anæsthetist would, if possible, be sought for when a shocked patient is to be operated on. This will often be impossible, and the following principles are laid down to help the less experienced anæsthetist :—

1. Do not start giving an anæsthetic unless the main anti-shock measures have already been started. Sometimes this is the duty of the anæsthetist, but in any case he should satisfy himself that the patient is being kept warm, that the intravenous infusion is worked satisfactorily, that movement of the patient is minimal, slow and gentle. He must know whether morphine has been given. He must see that whenever possible the patient's head is at shoulder level or lower.

2. The occasional anæsthetist should use the agent and method with which he is most familiar.

3. Anæsthesia for shocked patients is easily induced, and over-dosage is the common fault of the inexperienced. Shocked patients require far smaller quantities of any anæsthetic than the normal patient.

4. Oxygen is all important for these patients. An entirely unobstructed air-way must be maintained throughout, and additional oxygen is usually desirable.

Most general anæsthetics, while well tolerated in fit patients, are dangerous in patients gravely ill from shock, hæmorrhage or sepsis. The following considerations apply to such patients :—

*Ether.*—Ether anæsthesia may cause grave deterioration in the general condition of a shocked patient. Minimal quantities may be permissible to reinforce nitrous oxide and oxygen where the latter has proved inadequate. If nitrous oxide and oxygen are not available ether may have to be used alone. If so, the open method or Clover's inhaler are held to be undesirable and some apparatus such as the Oxford Vapouriser is advised. (If this is not available we presume Boyle's or Shipway's apparatus might be permitted.—Editor.)

*Chloroform.*—In the hands of an expert, chloroform may be satisfactory, but the sub-committee does not advise its general use. (This limited approval of chloroform is interesting.—Editor.)

*Vinyl Ether.*—Experience of this anæsthetic in shocked patients is small and the drug is in very short supply. The sub-committee thinks that perhaps vinyl ether, or a mixture of vinyl with ethyl ether may prove useful, either alone or combined with nitrous oxide and oxygen. (In any case the inexperienced anæsthetist is not likely to be called upon to use vinyl ether.—Editor.)

*Trichlorethylene.*—The sub-committee draws attention to the possibilities of trichlorethylene as an analgesic and anæsthetic in cases of shock and advises an early investigation of its qualities.

*Cyclopropane.*—This drug is in short supply and should not be used by the inexperienced except under expert supervision, and then probably only in special units. It has little deleterious effect on shocked patients. The high proportion of oxygen that can be used renders it specially suitable for patients suffering from severe anæmia, chest injuries, or respiratory embarrassment due to irritant gases.

*Nitrous Oxide.*—This should be used with oxygen, and provided anoxia is avoided does not appear to harm the shocked patient. Its range may be extended by the use of local anæsthesia. To the basal level of anæsthesia from nitrous oxide and oxygen may be added various adjuvants, of which the most suitable are ether, cyclopropane, pentothal or evipan. The sub-committee regard this method (with suitable apparatus such as is provided at all E.M.S. and many other hospitals) as the most appropriate for general adoption, and for the general instruction of learner-anæsthetists.

*Barbiturates.*—The sub-committee regards the short acting barbiturates (such as pentothal) as suitable for use on shock patients. These drugs, however, may easily prove lethal in careless or inexperienced hands. The dose needed is much reduced in shock or anæmic patients. The portability and simplicity of apparatus required renders the barbiturates especially suitable for use at dressing stations or field hospitals.

*Local Analgesia.*—The sub-committee is somewhat pessimistic on this subject. Further enquiry is necessary before the value of local analgesia can be judged. It must be shown to possess definite advantages before its general use could be recommended. There are considerable difficulties in the way of training an adequate number of anæsthetists in its use.

*Spinal Anæsthesia* should not be used where shock has supervened.

A few further notes are added to the sub-committee's recommendations by the Consultant Adviser in Anæsthetics.

*Morphine.*—Dosage for pain relief is frequently excessive in view of the patient's condition. Respiratory depression may prove dangerous during induction and maintenance of, as well as recovery from the anæsthetic. Allowance must be made for delayed absorption when the circulation is impaired. When a further dose of morphine is considered necessary, prior to induction of anæsthesia, it is best given slowly by intravenous injection and indilute solution.

*Carbon Dioxide.*—Persistent efforts to stimulate depressed respiration in shocked patients by $CO_2$ are more likely to do harm than good.

*Movement of Patients after Operation.*—The importance of slow and gentle movement of the patient from operating-table to bed should be impressed on nurses and orderlies.

*Oxygen Therapy.*—When immediate post-operative oxygen therapy is indicated, oxygen administration should begin on the operating-table and be continued during transfer to the ward.

It is interesting to compare the views expressed by Pender and Lundy in *War Medicine* (1942, 2, 193).

These authors, reviewing the topic of Anæsthesia in War Surgery, make the following predictions :—

1. Chloroform will be the reserve anæsthetic agent for use under adverse circumstances.

2. Ether will be the foundation anæsthetic agent when other agents are unsatisfactory.

3. Local anæsthesia will either be used alone or supplemented whenever possible.

4. Nitrous oxide and oxygen will be most useful for simple induction and for short operations.

5. Spinal anæsthesia administered by experienced hands will be safer than ever before for operations performed below the diaphragm.

6. Quick-acting barbiturates administered intravenously will be used more often than any other type of anæsthetic agent.

7. Rectal anæsthesia will seldom be used.

8. Cyclopropane will be unequalled for anæsthetizing many patients in base hospitals.

9. The intratracheal method will be almost the routine manner of administration of the volatile anæsthetic agents.

